# Public engagement in government officials’ posts on social media during coronavirus lockdown

**DOI:** 10.1371/journal.pone.0280889

**Published:** 2023-01-23

**Authors:** Ahmed Omar Bali, Hussam Al Halbusi, Araz Ramazan Ahmad, Ka Yiu Lee

**Affiliations:** 1 Diplomacy and Public Relations Department, University of Human Development, Sulaymaniah, Iraq; 2 Ahmed Bin Mohammed Military College, Doha, Qatar; 3 Department of Administration, College of Humanities, University of Raparin, Ranya, Iraq; 4 Department of International Relations & Diplomacy, Faculty of Administrative Sciences and Economics, Tishk International University, Erbil, Iraq; 5 Department of People and Society, Swedish University of Agricultural Sciences, Alnarp, Sweden; 6 Department of Health Sciences, Swedish Winter Sports Research Centre, Mid Sweden University, Östersund, Sweden; Universita degli Studi della Campania Luigi Vanvitelli, ITALY

## Abstract

**Background:**

Social media has been a common platform to disseminate health information by government officials during the COVID-19 pandemic. However, little is known about the determinants of public engagement in officials’ posts on social media, especially during lockdown.

**Objectives:**

This study aims to investigate how the public engages in officials’ posts about COVID-19 on social media and to identify factors influencing the levels of engagement.

**Methods:**

A total of 511 adults aged 18 or over completed an online questionnaire during lockdown in Iraq. Levels of engagement in officials’ posts on social media, trust in officials and compliance of government instructions were assessed.

**Results:**

Fear of COVID-19 and trust in officials were positively associated with compliance of government instructions. Trust in officials was also associated with active engagement in officials’ posts on social media, including commenting, posting and sharing of the posts.

**Conclusions:**

Trust in government has been established during the COVID-19 pandemic. Public engagement in officials’ posts is crucial to reinforce health policies and disseminate health information.

## Introduction

When the first case of coronavirus (COVID-19) was identified in Iraq on February 24, 2020 [1], government officials started to raise public awareness through social media and provide information and instructions about social distancing and hygienic practices [2]. One of the key features of social media is a two-way communication which allows government officials to deliver messages and to receive feedbacks from the public. Public confidence and trust could be gained in this process [3,4].

During the COVID-19 pandemic, people were encouraged to engage in officials’ posts on social media which may help raise health awareness of people [5]. In fact, social media has been shown to play a vital role on the outbreak of Zika [6–8], H1N1 [9], and Ebola [10]. Previous studies also found a positive role of social media on providing important health information [11–17]. In contrast, social media may also spread misinformation and trigger panics [18–25]. The participation of health care professionals on social media can reduce the possibility of fake news because they provide relevant and necessary information on medications, symptoms [26], disease management and diagnoses [27,28]. In addition, the input from health professionals can reduce fake news [29].

Social media has been found to connect people with government authorities in several studies with different approaches of analysis, including a descriptive content analysis of posts [6], social network analysis [9], thematic analysis [30], and comparative studies of countries in relation to health authorities [2]. However, there is a lack of research examining public engagement in officials’ posts about COVID-19 on social media, including reports, texts, pictures, videos, and interviews which are displayed by government officials.

Public engagement has become the center of attention in marketing, business, and communication technologies [31,32] which facilitate social interactions between parties during the crisis [33]. Social media offers two-way communication and encourages interactions among users through posting and sharing of texts, comments, pictures, and videos with a large audience anywhere at anytime [34]. Hwang and Thorn [35] categorized the public engagement into psychological and behavioral aspects. Psychological engagement refers to personal and internal involvement in posted texts, videos, and data in a specific context. Behavioral engagement is achieved when the users click “like”, write comments and posts, or share a text, picture, or video with the public or with a close group on social media [33].

Several studies revealed that that people engage more with social media during health crises and pandemics [36]. For example, the majority of Chinese were engaged in social media during lockdown [37,38]. Deng et al. [24] explained that the “online engagement and psychological distance” are the reasons people engaging in social media during the crisis. Social media also raises awareness of crisis and helps connection with family and friends. Liu, et al [39] explained the importance of “psychological distance” through analyzing the behaviors of internet users and found that quarantine policies increase the public anxiety due to social distancing. Few studies, however, examine the levels of fear of COVID-19 during lockdown period and the relationship with public engagement in social media [40,41].

Social media enables users to receive immediate information which can be shared and discussed among friends and relatives [42,43]. It is expected that information exchange between people will increase during the crisis, particularly during lockdown when people lose physical interactions. However, there is a lack of research examining the determinants of public engagement in officials’ posts during lockdown. This study, therefore, serves to fill in the above research gaps.

## Methods

This is a cross-sectional study conducted through a self-administered online questionnaire targeting adults aged 18 years or above in Iraq. The questionnaire measures levels of public trust in government officials, engagement in officials’ posts on social media and other health behaviours based on a binary scale (Yes/No) or a 3-point scale (Yes/To somewhat/No). The online questionnaire was distributed using social media, including Facebook, Viber, Instagram and WhatsApp. Data collection was carried out during the lockdown period in Iraq starting from the first week of March until the mid of April 2020. Informed consent was obtained from participants and approval to conduct this study was obtained from the University of Raparin.

### Statistical analysis

Descriptive statistics of key variables were presented as frequency and percentage. Spearman correlation tests were conducted to examine the association between public engagement in social media, trust in government officials and other health behaviours. Chi-square tests were used to determine the frequency of categorical variables. All data analyses were conducted using IBM SPSS version 22.

## Results

A total of 511 participants completed the questionnaire, of which 313 (61.3%) participants were male. Over 61% (n = 314) participants were 18–35 years old, whilst about 30% (n = 154) participants were 36–50 years old.

### Public trust in government officials

The majority of participants (62.1%) indicated that they somewhat trust the national healthcare, whilst 12.7% participants indicated their distrust. A total of 71.8% participants indicated their trust in the instructions given by officials on social media and over 95% participants believed in government strategies to prevent spreading of COVID-19, such as social distancing and mask wearing. Most participants (65.9%) indicated that they somewhat fear of COVID-19 (Table 1).

**Table 1:** Public trust in national healthcare, instructions given by officials on social media, government strategies to prevent spreading of COVID-19, and fear of COVID-19 among 511 participants.

Variables	N (%)
**Public trust in national healthcare**	
No	56 (12.7)
To somewhat	312 (62.1)
Yes	132 (26.2)
**Public trust in the instructions presented on social media by government officials**	
No	54 (10.6)
To somewhat	90 (17.6)
Yes	367 (71.8)
**Believing in government strategies to prevent spreading of COVID-19 (Mask wearing, social distancing, etc.)**	
No	23 (4.5)
Yes	488 (95.5)
**Fear of COVID-19**	
No	70 (13.7)
To somewhat	337 (65.9)
Yes	104 (20.4)

### Public engagement in officials’ posts and behavioural change

The majority of participants (61.1%) indicated that they somewhat wrote comments and clicked like on the officials’ posts about COVID-19 on social media, while about one-fifth of the participants did not react to the officials’ posts. Similarly, over half of the participants indicated that they somewhat posted and shared officials’ posts, whilst about 38% participants did not. Over 55% participants investigated the information about COVID-19 on social media according to the information from international sources, whilst nearly 84% participants changed their behaviours in response to officials’ posts about COVID-19 (Table 2).

**Table 2:** Public engagement in officials’ posts about COVID-19 on social media and behavioural change in response to officials’ posts among 511 participants.

Variables	N (%)
**Writing comments and clicking like in officials’ posts about COVID-19 on social media**	
No	112 (21.9)
To somewhat	312 (61.1)
Yes	87 (17.0)
**Posting and/or sharing officials’ posts about COVID-19 on social media**	
No	191 (38.2)
To somewhat	271 (53.0)
Yes	45 (8.8)
**Investigating the information about COVID-19 on social media according to the information from international sources**	
No	228 (44.6)
Yes	283 (55.4)
**Changing behaviour in response to officials’ posts about COVID-19 on social media**	
No	81 (15.9)
Yes	430 (84.1)

### Public engagements in officials’ posts between sexes and age groups

More male participants than female indicated that they clicked like or commented on the officials’ posts on social media (X^2^ = 7.79, p < .05). Similarly, more male participants shared officials’ posts (X^2^ = 11.54, p < .05) (Table 3). More participants in younger age group (18–35 years old) had somewhat clicked like or commented on officials’ posts (X^2^ = 20.38, p < .05), whilst more participants in older age group indicated that they shared the officials’ posts (X^2^ = 19.23, p < .05).

**Table 3:** Differences in public engagements in officials’ posts between sexes and age groups *(N = 511)*.

Gender	Levels of Engagement	X^2^	p-value	No (N (%))	To some what (N (%))	Yes (N (%))
*Male*	Liking or commenting on a post	7.79	.005	61(19.5)	187(59.7)	65(20.8)
*Female*				51(25.8)	125(61.3)	22(11.1)
*Male*	Sharing of a post	11.54	.003	102 (32.6)	178 (59.9)	33 (10.5)
*Female*				93 (47)	93 (47)	12(6.1)
**Age (years)**						
*18–35*	Liking or commenting on a post	20.38	.000	54(17.2)	213(67.8)	47(15)
*36–50*				42(27.3)	83(53.9)	29(18.8)
*51 or above*				16(37.2)	16(37.2)	11(25.6)
*18–35*	Sharing of a post	19.23	.001	118(37.6)	155(49.4)	41(13.1)
*36–50*				59(38.3)	91(59.1)	4(2.6)
*51 or above*				18(14.9)	25(58.1)	0 (0)

### Fear of COVID-19, compliance of government instructions and public engagement in officials’ posts on social media

There was significant association between fear of COVID-19 and compliance of government instructions (rho = .183, p < .000). There was also significant association between trust in officials’ posts and compliance of government instructions (rho = .294, p < .000), clicking like and/or writing comments (rho = 0.181, p < .000), and sharing the officials’ posts (rho = .129, p < .003), indicating that trust in government helps disseminate health information and reinforce health policies.

## Discussion

Iraqi government has lost public trust due to corruption [44] and insufficient public health services [31]. In addition, the media is monopolized by certain political parties with different ethnic and religious groups, causing public polarization [34,45]. The government officials tend to be acceptable only among a certain group [46,47], and they have been under criticism when publishing posts on social media, especially from the opposition groups who do not often use social media. However, during the COVID-19 pandemic, particularly during the lockdown, government officials were viewed as active members and the public engagement in officials’ posts on social media was positive. In this study, the young male group showed more engagement in officials’ posts. It was also found that the majority of the public trusted the officials’ posts about COVID-19 on social media. We revealed that the fear of COVID-19 had changed their perception of the government authorities. Natural crisis can be a good opportunity for restoring public confidence and trust in government and creating relationships between the public and officials. Our results found that constructive dialogues between the public and officials can facilitate the interaction and play a role in mitigating the crisis. In this context, our study found that the majority of participants clicked like and wrote comments on the officials’ posts about COVID-19 on social media, whilst less participants posted and shared the officials’ posts. This indicates that sharing of a post involves a more complex cognitive process [32]. Social distancing is a factor of active engagement in social media as the need to interact with others surges during the lockdown [36–39]. It is worth noticing that only 26.2% of the participants indicated that they trust the national healthcare. This could be explained by the fact that Iraqi health system is not updated and corrupted. In addition, medical and academic professionals are not financially supported by the government [48–52]. In summary, public engagement in the media context includes the communication and understanding between specialists and professionals and the ordinary public, which contributes to building up a scientifically literate society [53], particularly in the developing world [54] through increasing their health awareness. In addition, it helps the health sectors and officials in decision-making regarding outbreak management [55].

### Limitations

Although internet access was free of charge during the data collection period, younger and more educated groups are more likely to access to internet and participate in this study, causing certain extent of sampling bias. The cross-section study design implies that no causal relationship can be drawn. In addition, this study relied on self-report data which could be subjected to recall bias.

## Conclusion

This study found that public trust in government authority was regained during the COVID-19 pandemic. There were correlations between fear of COVID-19, compliance of government instructions and public engagement in officials’ posts on social media. Public engagement in officials’ posts has played a role in disseminating health information and preventing the spread of COVID-19.

## Supporting information

S1 File (SAV)
